# Abnormal myocardial enzymes are important indicators of poor prognosis in COVID-19 patients

**DOI:** 10.2217/fvl-2020-0304

**Published:** 2021-04-07

**Authors:** Jingping Cheng, Wanxin Liu, Siyang Chen, Xiafen Hu, Xiaochen Xiang, Zhongliang Cheng, Shaoqian Cai, Kaiwen Guo, Qiang Wang, Xiaoliu Liu, Qingming Wu

**Affiliations:** 1 ^1^Department of Gastroenterology, CR & WISCO General Hospital, Wuhan University of Science & Technology, Wuhan, Hubei, China; 2 ^2^Institute of Infection, Immunology & Tumor Microenvironment, Hubei Province Key Laboratory of Occupational Hazard Identification & Control, Medical College, Wuhan University of Science & Technology, Wuhan 430065, Hubei, China

**Keywords:** CK, COVID-19, LDH, prognostic evaluation, α-HBDH

## Abstract

**Objective:** Researching the prognostic value of myocardial enzymes in COVID-19 patients. **Materials & methods:** We collected 113 confirmed COVID-19 patients. The dynamic changes of CK, LDH and α-HBDH in patients were studied retrospectively, the correlation between myocardial enzyme index, clinical classification and outcome of patients and its significance to prognosis. **Results:** There are significant statistical differences between LDH, α-HBDH, CK and the clinical classification, and patient’s outcome. In the receiver operating characteristic curve analysis, LDH, α-HBDH and CK have a good diagnostic value for the death outcome of patients. **Conclusion:** LDH, α-HBDH and CK were the components of myocardial enzyme profiles, and our results found that they were significantly positively correlated with clinical classification and prognosis of COVID-19 patients. The values of LDH, α-HBDH and CK increased with the increase of the severity of admission clinical classification and the deterioration of outcome. Therefore, we propose that continuous monitoring of LDH, α-HBDH and CK indicators can warn the deterioration of COVID-19 to a certain extent, regardless of whether patients with cardiovascular diseases are combined or not, and prompt early intervention.

In December 2019, novel coronavirus was found in Wuhan, China. The International Committee on Taxonomy of Viruses named it severe acute respiratory syndrome coronavirus 2 (SARS-CoV-2), and the pneumonia caused by the virus was officially named corona virus disease (COVID-19) by the WHO. COVID-19 has caused a pandemic worldwide and become a public health problem that endangers human health. The main clinical manifestations of COVID-19 patients are respiratory damage. Most patients have a good prognosis, but some patients have varying degrees of myocardial impairment, their condition changes rapidly and even threaten the life. Therefore, it is very important to judge the severity and prognosis of COVID-19 patients in the early stage. The laboratory indicators of myocardial enzymes were included in this research: LDH (or LD), α-HBDH, CK. Are these indicators also of diagnostic value to patients with COVID-19? To this end, we researched the dynamic changes of LDH, α-HBDH and CK in peripheral blood of COVID-19 patients to evaluate whether they can be used as the severity and prognosis of COVID-19 patients.

## Materials & methods

This is a retrospective observational study. All the cases were collected from CR & WISCO General Hospital affiliated to Wuhan University of Science and Technology, Wuhan city, Hubei province, China. They were admitted to the hospital from 12 January 2020 to 19 February 2020, and were positive for SARS-CoV-2 nucleic acid test.

### Sample inclusion criteria

Patients who have completed laboratory data collection and clinical assessment at three time points required for this research. The patients in the research were cured, discharged or died before 23 March 2020; the duration of hospitalization was greater than 10 days for patients who were treated in hospital on 23 March 2020.

### Exclusion criteria

Incomplete laboratory data collection for three-times; at the composite end point of the study, those who had been hospitalized for less than 10 days on 23 March 2020.

### Clinical evaluation criteria

#### Case classification

According to the diagnosis and treatment plan of COVID-19 issued by the National Health Commission of China, it was divided into four types: mild, ordinary, severe and critically severe. In this study, the mild type and the ordinary type were combined into one type.

Mild type: the clinical symptoms were mild, and no signs of pneumonia were found on computed tomography (CT) imaging.

Ordinary type: the patients had fever and respiratory symptoms, and CT images that showed signs of pneumonia.

Severe type: satisfy any of the following:

- Shortness of breath appeared, respiratory rate (RR) >30-times/min;
- At rest, oxygen saturation ≤93%;
- PaO2/ FiO2 ≤ 30 mmHg (1 mmHg = 0.133 kPa), pulmonary imaging showed that the lesion progressed to >50% within 24–48 h.

Critically severe type: patients who meet one of the following conditions:

- Respiratory failure occurs and mechanical ventilation is required;
- The patient developed symptoms of shock;
- ICU care is required for patients with other organ failure.

The purpose of this research was to evaluate the role of peripheral blood inflammatory indicators in the assessment of severity and prognosis of COVID-19.

#### Classification of CT images

According to the diagnosis and treatment protocol, the CT images were divided into normal, mild, progressive and severe.

Mild type: the main performance is the size of unequal, focal ground glass shadow. In some cases, there are small patchy ground glass shadows, subpleural ground glass shadows or ground glass nodules and subsolid nodule. The lesions can be multiple or single, and can be involved in all lung lobes. The lesions are mostly seen in the middle and lower lobes, and are mostly distributed in the peripherals and subpleural areas of the lung. The main manifestations are ground glass shadows and consolidation shadows.

Progressive type: the range of lesions is large, often involving multiple pulmonary lobes of both lungs. Consolidation and fibrosis of different sizes and degrees occur in the lesions. Multiple lesions coexist, which may be accompanied by tractive bronchial or bronchiectasis, localized interlobar pleural thickening and rare enlargement of pleural effusion and mediastinal lymph nodes.

Severe type: the lesions of the two lungs are diffuse and uneven in density, and large areas of consolidation can be seen, or large pieces of ground glass shadow can be seen, and air bronchial signs can be seen therein. ‘White lung’ was seen when most of the lungs were involved. The diaphragmatic surface was elevated and the interlobar pleura and bilateral pleura were thickened, with a small amount of pleural effusion.

#### The disease outcome

According to the clinical characteristics, the patients were divided into three types: discharge, improvement and death. This project was approved by the medical ethics committee of Wuhan University of Science and Technology, the ethical code is 202009.

### Therapeutic method

According to the COVID-19 diagnosis and treatment plan issued by the National Health Commission of China, patients generally received effective oxygen therapy, lopinavir/ritonavir antiviral treatments and provide respiratory and circulatory support treatment for patients with severe and critically severe illness.

### Study on composite end point

By 23 March 2020, we counted the number of cases of discharge and death before 23 March 2020, and then included the patients who had been in hospital for more than 10 days and evaluated the changes of their conditions, which were determined as discharge, improvement and death.

### The collection of experimental data

The researchers collected data at three time points: at admission, 3–5 days in hospital, and at the end of the illness. During the study, LDH, CK and α-HBDH of peripheral blood were collected, and the clinical typing, severity and outcome of the patients were evaluated. Meanwhile, the corresponding pulmonary CT imaging data were collected.

### Statistical method

SPSS 25.0 version of the software is used for statistical analysis. The mean (M) ± standard deviation (SD) was described in the measurement data, and the differences between the groups were analyzed by the multivariate analysis of variance and the chi-square test of independent samples if p < 0.05 was considered statistically significant. The receiver operating characteristic curve (ROC) analysis calculated the levels of LDH, CK and α-HBDH to determine the area under the curve (AUC) and 95% CI of COVID-19 patients, providing both sensitivity and specificity. Spearman correlation coefficient was used in the correlation analysis to measure the correlation degree of the hierarchical and sequenced variables in this study. The test level selected for this statistical analysis is α = 0.05; p < 0.05 indicates that the difference is statistically significant.

## Result

### Demographic characteristics

During 12 January 2020 at solstice 19 February, a total of 113 COVID-19 cases were collected from CR & WISCO General Hospital affiliated to Wuhan University of Science and Technology and met the requirements of this research. The sample ages ranged from 29 to 92 years, with an average age of 63 years; 53 of them were male, accounting for 46.9%, 60 were female, accounting for 53.1%, and 73 were over 60, accounting for 64.6%. In the first clinical classification of the patients at admission, 72 patients were mild (63.7%), 18 patients were severe (15.9%), and 23 patients were critically severe (20.4%). There were more males than females, and more patients over 60 years old than patients under 60 years old. The age of severe, critically severe and dead patients was older, which was consistent with the literature reports [1].

**Figure 1.**
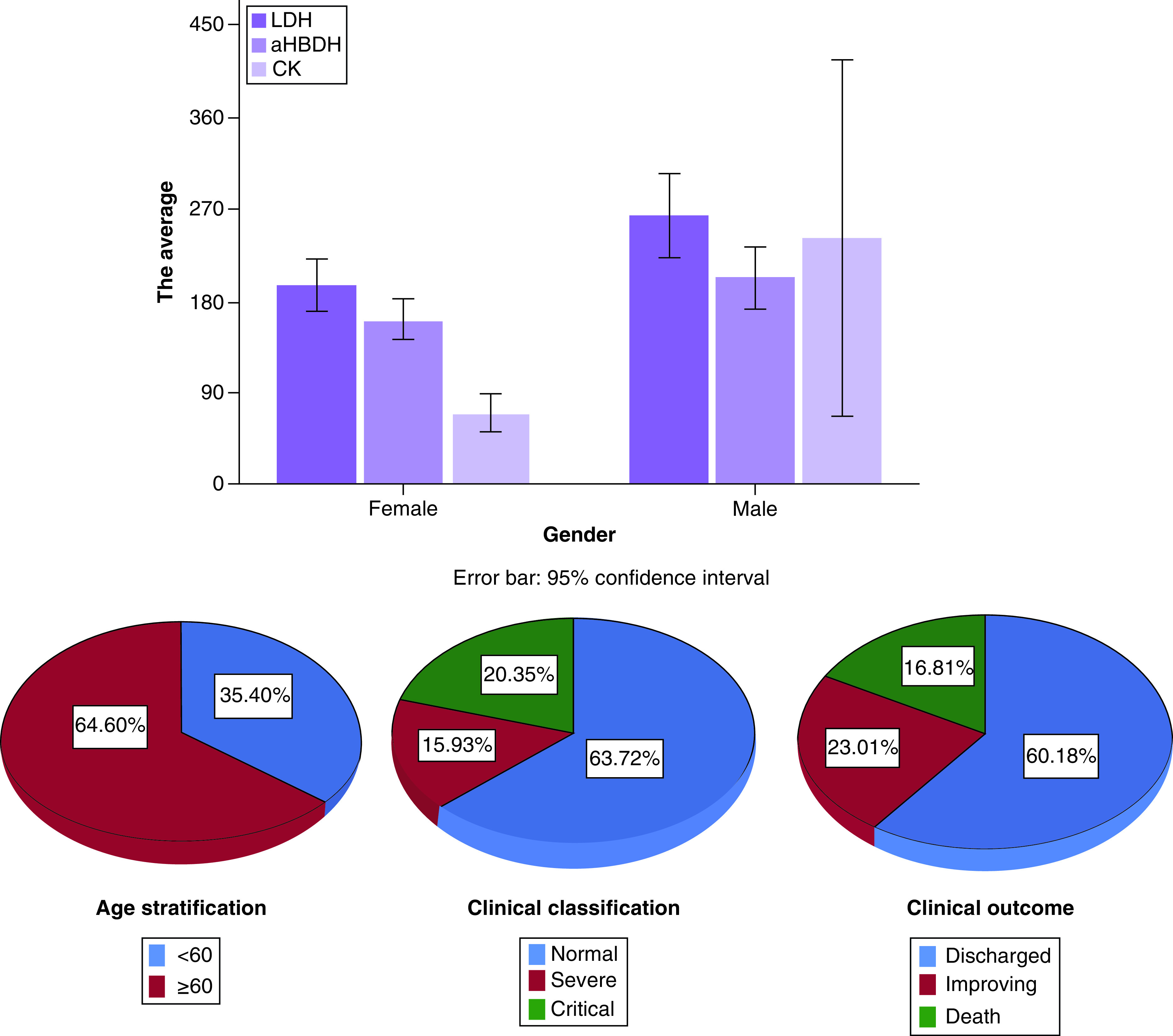
The relationship between age, gender and clinical typing at admission and outcome. This research included LDH, α-HBDH, CK to explore the myocardial function damage of COVID-19 patients. The mean (M) ± standard deviation (SD) of LDH was 226.77 ± 128.21, and the average value of male patients (263.00 ± 148.83) was higher than that of female patients (194.77 ± 97.25). The M ± SD of α-HBDH is 180.85 ± 94.25, and the mean value of male patients (202.57 ± 109.26) is higher than that of female patients (161.67 ± 74.47). The M ± SD of CK was 149.92 ± 445.84, and the mean value of male patients (241.19 ± 637.33) was higher than that of female patients (69.30 ± 72.43). The average age of the included patients was 63 years old, with 73 patients over 60 years old accounting for 64.6%, and 40 patients under 60 years old accounting for 35.4%. In the first clinical classification of the patients at admission, 72 patients were mild (63.7%), 18 patients were severe (15.9%) and 23 patients were critically severe (20.4%). The outcomes of all patients were divided into discharge, improving and death, among which discharge was the most common outcome (n = 68), accounting for 60.18%, death was less common (n = 19), accounting for 16.81%, and the condition of some inpatients was improved (n = 26), accounting for 23.01%.

The demographic and clinical characteristics of 113 COVID-19 positive patients are shown in Table 1. The average age of patients included in the research was 63 years old, with the average age of male patients (65 years old) being higher than that of female patients (60 years old). As the cut-off point, patients aged 60 years were divided into two groups: those aged above 60 years and those aged below 60 years. The number of patients aged 60 years and above was significantly higher than that of patients aged below 60 years. The clinical classification of the diagnosed patients at admission was based on CT scores, which were divided into mild, severe and critically severe. In this study, the majority of patients were mild type (n = 72), severe type (n = 18) and critically severe type (n = 23). Compared with female patients, there were more severe and critically severe patients in male patients. The outcomes of all patients were divided into discharge, improvement and death. Among them, the majority of patients were discharge outcomes (n = 68), few patients died (n = 19) and some inpatients showed improvement (n = 26). Between sexes, more men died than women and more women were discharged and improved. This study included LDH, α-HBDH, CK and other indicators to explore the myocardial function damage of COVID-19 patients. The M ± SD of LDH was 226.77 ± 128.21, and the average value of male patients (263.00 ± 148.83) was higher than that of female patients (194.77 ± 97.25). The M ± SD of α-HBDH is 180.85 ± 94.25, and the mean value of male patients (202.57 ± 109.26) is higher than that of female patients (161.67 ± 74.47). The M ± SD of CK was 149.92 ± 445.84, and the mean value of male patients (241.19 ± 637.33) was higher than that of female patients (69.30 ± 72.43) (see Table 1 & Figure 1 for details).

**Table 1.** Demographic and clinical characteristics.

	Male, mean (SD)	Female, mean (SD)	Overall, mean (SD)
Age (years)	65 (13.8)	60 (13.2)	63 (13.62)
<60	13	27	40
≥60	40	33	73
HOD	17.1 (4.60)	16.2 (3.74)	16.5 (4.06)
**Clinical characteristics**			
Mild type	26	46	72
Severe type	12	6	18
Critically severe	15	8	23
**Outcome**			
Discharge	31	37	68
Improvement	9	17	26
Death	13	6	19
LDH (mg/l)	263.00 (148.83)	194.77 (97.25)	226.77 (128.21)
109–245	31	54	85
<109	1	0	1
>245	21	6	27
α-HBDH (l)	202.57 (109.26)	161.67 (74.47)	180.85 (94.25)
72–182	31	51	82
<72	0	0	0
>182	22	9	31
CK (l)	241.19 (637.33)	69.30 (72.43)	149.92 (445.84)
38–174	37	38	75
<38	5	19	24
>174	11	3	14

Normal reference values: LDH (<3 mg/l); α-HBDH (1.1 × 10^9^/l – 3.2 × 10^9^/l); CK(1.1 × 10^9^/l – 3.2 × 10^9^/l); HOD: Hospitalization days.

All patients were classified according to clinical severity when they were admitted. They were mild, severe and critically severe. In the analysis of the differences between age, gender with clinical typing and outcome after admission, the age of 60 was taken as the threshold and set as the dichotomous variable, then, the relationship between age, gender with clinical typing and outcome was investigated by bilateral chi-square test according to the variable type, if the theoretical frequency was less than 1, the Fisher exact probability test was used. The results showed that there was a significant statistical difference between gender and admission clinical typing, and there was a significant statistical difference between age and outcome (see Table 2 for details).

**Table 2.** The relationship between age, gender and clinical typing at admission and outcome.

Characteristic	Clinical typing at admission					
	Mild	Severe	Critically severe	Total	χ^2^	p-value
Age^†^ (years)						
<60	29	7	4	40	4.107	0.128
≥60	43	11	19	73		
Total	72	18	23	113		
Gender^†^						
Male	26	12	15	53	9.288^¶^	0.009
Female	46	6	8	60		
Total	72	18	23	113		

Characteristic	Outcome					
	Mild	Severe	Critically severe	Total	χ^2^	p-value
Age^†^ (years)						
<60	23	15	2	40	10.865^¶^	0.005
≥60	45	11	17	73		
Total	68	26	19	113		
Gender^†^						
Male	31	9	13	53	5.156	0.076
Female	37	17	6	60		
Total	68	26	19	113		

† Chi-square test (double-tail test).

¶ At level 0.05 (double-tailed), the correlation was significant.

As shown in Table 3, LDH, α-HBDH, CK gradually increased with the increasing severity of clinical typing, and the mean values of LDH, α-HBDH and CK in severe and critically severe patients were higher than the mean of all samples. We used the chi-square test (if the theoretical frequency is less than 1, Fisher’s exact probability-test method) to find that there were significant statistical differences between LDH, α-HBDH, CK and clinical typing (p < 0.05). It shows that the changes of LDH, α-HBDH and CK have a correlation with the clinical classification of patients on admission.

**Table 3.** The relationship between LDH, α-HBDH, CK and clinical typing.

Clinical typing	^‡^LDH (U/l)					^†^α-HBDH (U/l)				
	n	$\bar{\text{x}}$ ± s	<109 (n)	109–245	>245 (n)	n	$\bar{\text{x}}$ ± s	<72 (n)	72–182	>182 (n)
Mild	72	171.78 ± 36.80	1	68	3	72	142.42 ± 29.14	0	67	5
Severe	18	296.00 ± 97.71	0	7	11	18	233.56 ± 74.75	0	5	13
Critically severe	23	344.74 ± 208.71	0	10	13	23	259.91 ± 156.60	0	10	13
Total	113	226.77 ± 128.21	1	85	27	113	180.85 ± 94.25	0	82	31
χ^2^/p	χ^2^ = 45.60^¶^		p = 0.000			χ^2^ = 42.88^¶^		p = 0.000		

Clinical typing	^†^CK (U/l)									
	n	$\bar{\text{x}}$ ± s	<38 (n)	38–174	>174 (n)					
Mild	72	57.18 ± 32.64	19	53	0					
Severe	18	180.89 ± 172.34	3	9	6					
critically severe	23	416.00 ± 939.47	2	13	8					
Total	113	149.92 ± 445.84	24	75	14					
χ^2^/p	χ^2^ = 29.01^¶^		p = 0.000							

† Chi-square test (double-tail test);

‡ Fisher’s exact test (double-tail test).

¶ At level 0.05 (double-tailed), the correlation was significant.

As can be seen from Table 4, LDH, α-HBDH and CK also increased with the deterioration of outcome (discharge, improvement and death). We used the chi-square test (Fisher’s exact probability test was used if the theoretical frequency was less than 1 to analyze and found that there were significant statistical differences between LDH, α-HBDH, CK and the outcome (p < 0.05). It indicated that the changes of LDH, α-HBDH, CK were correlated with the outcome of patients.

**Table 4.** Relationship between LDH, α-HBDH, CK and outcome.

Outcome	^‡^LDH (U/l)					^†^α-HBDH (U/l)				
	n	$\bar{\text{x}}$ ± s	<109 (n)	109–245	>245 (n)	n	$\bar{\text{x}}$ ± s	<72 (n)	72–182	>182 (n)
Discharged	68	222.07 ± 126.30	1	53	14	68	179.16 ± 98.67	0	51	17
Improve	26	170.65 ± 38.02	0	24	2	23	145.88 ± 29.55	0	23	3
Death	19	320.37 ± 164.34	0	8	11	19	234.74 ± 113.93	0	8	11
Total	113	226.77 ± 128.21	1	85	27	113	180.85 ± 94.25	0	82	31
χ^2^/p	χ^2^ = 15.55^¶^		p = 0.001			χ^2^ = 12.36^¶^		p = 0.002		

Outcome	^†^CK (U/l)									
	n	$\bar{\text{x}}$ ± s	<38 (n)	38–174	>174 (n)					
Discharged	68	125.19 ± 286.42	15	45	8					
Improve	26	59.62 ± 34.25	7	19	0					
Death	19	361.99 ± 931.81	2	11	6					
Total	113	149.92 ± 445.84	24	75	14					
χ^2^/p	χ^2^ = 10.72^§^		p = 0.028							

† Chi-square test (double-tail test);

‡ Fisher’s exact test (double-tail test).

§ At level 0.05 (double-tailed).

¶ At level 0.05 (double-tailed), the correlation was significant.

**Figure 2.**
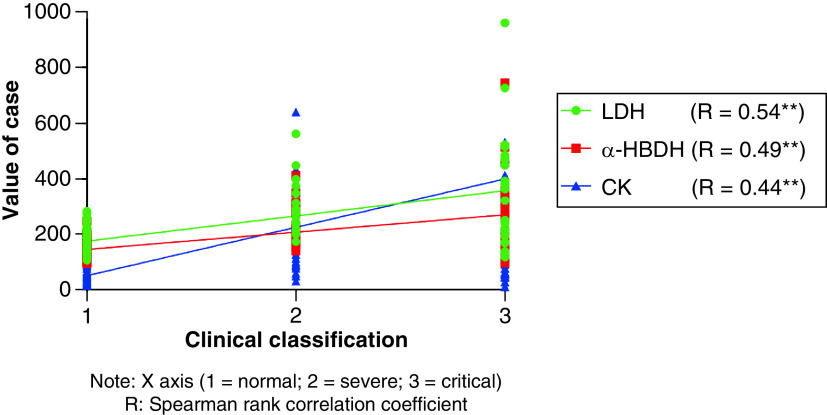
Correlation analysis between LDH, α-HBDH, CK and clinical typing. Spearman rank correlation analysis was used to analyze the correlation between LDH, α-HBDH, CK and clinical classification (1 = normal, 2 = severe, 3 = critical). The results showed that LDH, α-HBDH, CK were positively correlated with clinical classification at admission (p < 0.05), and the correlation coefficients were LDH 0.54, α-HBDH 0.49, CK 0.44.

**Figure 3.**
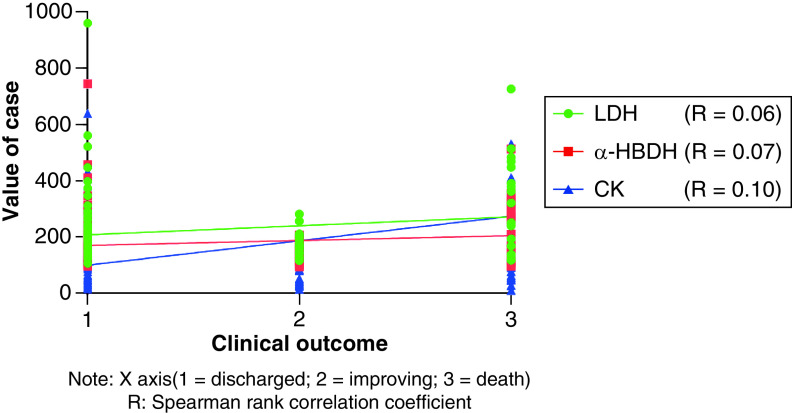
Correlation analysis between LDH, α-HBDH, CK and the outcome. Spearman rank correlation analysis was used to analyze the correlation between LDH, α-HBDH, CK and outcome (1 = discharge, 2 = improving, 3 = death), and the results showed that there was no significant correlation between LDH, α-HBDH, CK and outcome.

Clinical classification and outcome are both grade variables. Therefore, Spearman rank correlation analysis was adopted in Figures 2 and 3 to research the relationship between LDH, α-HBDH, CK and clinical typing and outcome. The results showed that LDH, α-HBDH and CK were positively correlated with clinical typing at admission (p < 0.05), and the correlation coefficients were 0.54, 0.49 and 0.44, respectively. There was no significant correlation between LDH, α-HBDH, CK and outcome.

**Figure 4.**
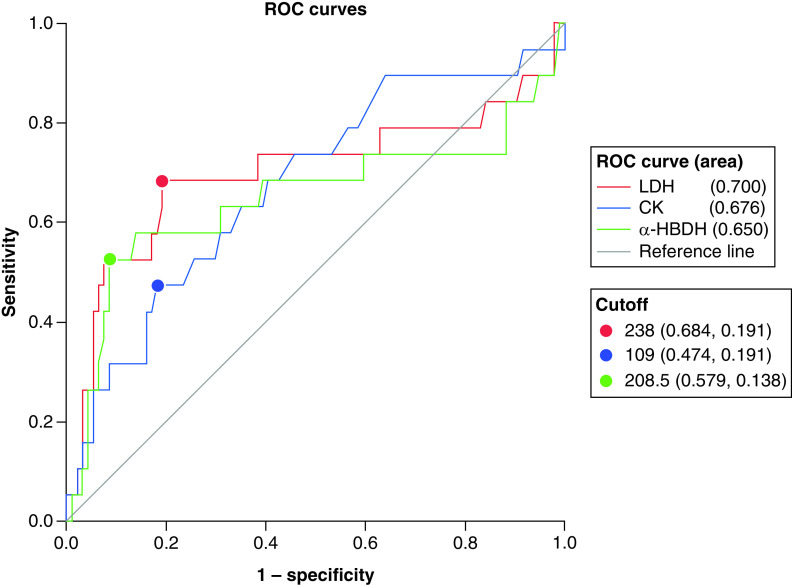
Diagnostic value of LDH, α-HBDH and CK on the patient’s death outcome. In ROC curve analysis, we analyzed the diagnostic accuracy of LDH, α-HBDH and CK in differentiating the death outcomes of COVID-19 patients based on the death outcomes of the patients. The results showed that the AUC for differentiating the death outcomes of COVID-19 patients was from large to small: LDH > CK > α-HBDH, the specific values were 0.700 (95% CI: 0.536∼0.864; p < 0.01); 0.676 (95% CI: 0.536∼0.817; p < 0.05); 0.650 (95% CI: 0.476∼0.824; p < 0.05). The AUC of all three indicators was close to 0.7. LDH, α-HBDH and CK have a good judgment accuracy for the death outcome of COVID-19. The best cut-off values calculated by the maximum Youden index are 238, 208.5 and 109. AUC: Area under the curve; ROC: Receiver operating characteristic curve.

In ROC curve analysis, we analyzed the diagnostic accuracy of LDH, α-HBDH and CK in differentiating the death outcomes of COVID-19 patients based on the death outcomes of the patients. The results showed that the AUC for differentiating the death outcomes of COVID-19 patients was from large to small: LDH > CK > α-HBDH, the specific values were 0.700 (95% CI: 0.536∼0.864; p < 0.01); 0.676 (95% CI: 0.536∼0.817; p < 0.05); 0.650 (95% CI: 0.476∼0.824; p < 0.05). The AUC of all three indicators was close to 0.7. LDH, α-HBDH and CK have a good judgment accuracy for the death outcome of COVID-19. The best cut-off values calculated by the maximum Youden index are 238, 208.5 and 109, respectively (see Figure 4 for details).

**Figure 5.**
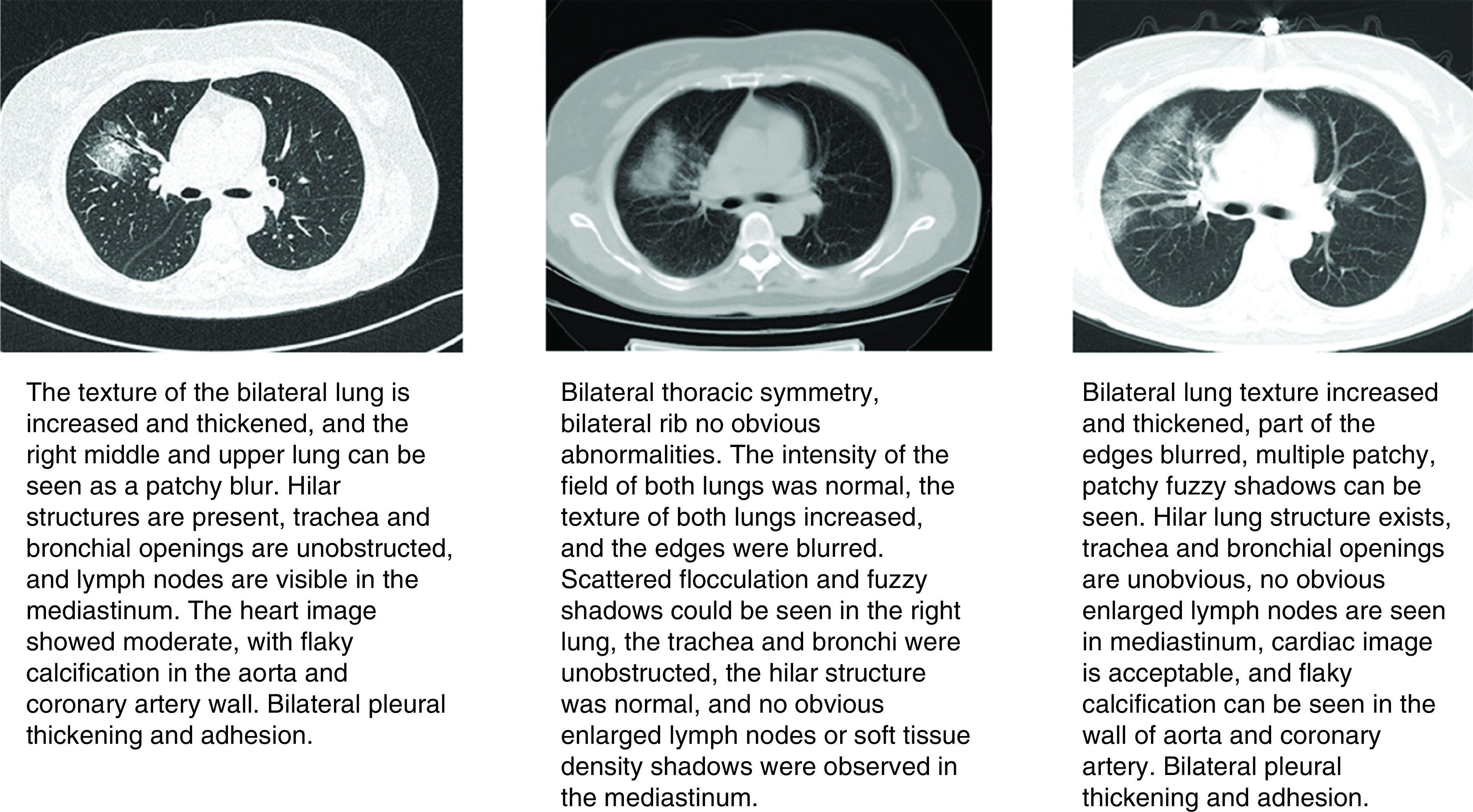
Dynamic changes of chest computed tomography of a patient. In this research, a female patient was selected for the third stage chest imaging (admission, the 3rd day of hospitalization, the end point), and the imaging conclusions suggested that the patient had right lung infection and associated atherosclerotic lesions. The dynamic changes of LDH, α-HBDH and CK related to myocardial injury were consistent with the results of chest computed tomography images.

The clinical observation of the researchers found that the abnormal results of myocardial enzyme spectrum were consistent with the results of CT imaging. In this research, a female COVID-19 patient aged over 60 years was selected as an example. The dynamic changes of chest CT images are described in Figure 5.

## Discussion

Initial symptoms of COVID-19 include fever, cough, strength, muscle soreness, sore throat and diarrhea, with fever as the primary symptom. Most of the patients were mild and a few were critically severe. Some patients may gradually develop dyspnea, and those in critically severe condition may progress rapidly and develop severe inflammatory storms resulting in death [2,3].

In addition to typical respiratory manifestations, a proportion of COVID-19 patients have clinical manifestations of cardiac involvement. An article published in the *Lancet* in 41 patients with confirmed COVID-19, according to the analysis shows that six patients (15%) with hypertension, six patients (15%) with cardiovascular diseases, five cases (12%) in patients with acute myocardial injury after infection and four cases into ICU treatment, so to a certain extent, shows the acute myocardial injury is associated with the progress of the disease [4]; Another study published in the *Lancet* included 99 patients with COVID-19, more than half of whom (51%) had a history of chronic disease, and up to 40 (40%) had a history of cardiovascular and cerebrovascular disease. Myocardial enzyme showed that most of the patients had abnormal myocardial enzyme, 13 (13%) of which had creatine kinase elevated and 75 (76%) of which had lactate dehydrogenase elevated. Therefore, it is speculated that COVID-19 infection is associated with complicated cardiovascular disease [3,5]. Based on a large number of COVID-19 patients with combined cardiovascular system symptoms [6,7], and accompanied by the phenomenon of high mortality in patients with cardiovascular diseases [8], ‘COVID-19 therapy (trial version 7)’ and ‘COVID-19 heavy, severe cases of diagnosis and treatment plan (trial version 2)’ clearly suggested according to condition monitoring lactate dehydrogenase, myocardial injury markers [9,10].

Clinical data of COVID-19 patients showed [11] that lactate dehydrogenase, creatine kinase and myoglobin were elevated; troponin was elevated in some critically ill patients. W Chaomin professor and his team have analyzed the heart damage markers associated with mortality and death time relations, including hypersensitive troponin I (hs - TNI), CK, CK isoenzyme (CK-MB), LDH and α-HBDH, the results indicate that cardiac injury signs and COVID-19 patients with high mortality and earlier death [12].

The myocardial enzyme spectrum in clinical detection mainly includes five components: glutamate aminotransferase, CK, CK isoenzyme (ck-mb), LDH and α-HBDH. CK [13] is also known as CPK. It is mainly found in the cytoplasm and mitochondria, and is most abundant in skeletal muscle and cardiac muscle, followed by brain tissue and smooth muscle, and is less abundant in liver, pancreas and red blood cells. CK is a dimer composed of M-type and B-type subunits, which forms three isomeric isozymes, namely CK-MM, CK-MB, CK-BB. CK-MB type is the most abundant in myocardium, CK-MM Type in skeletal muscle and CK-BB Type in brain. LDH [14] is an important enzyme in energy metabolism in the body. It is found in almost all tissues, especially the liver, kidney, heart muscle, skeletal muscle, pancreas and lungs. LDH is often used in the diagnosis of myocardial infarction, liver disease and some malignant tumors. α-HBDH [15] is not an independent specific enzyme, but a general term for LD-1 and LD-2 containing H subunits. It is synthesized from the mitochondria of hepatocytes and is widely distributed in the body. The activity of lactate dehydrogenase isoenzymes LDH1 and LDH2 was actually reflected in the determination of α-HBDH, which was valuable for the diagnosis of myocardial disease and liver disease.

CK, LDH and α-HBDH are partial indicators of myocardial enzyme spectrum. So, we put forward the research hypothesis, as a newly emerging acute infectious disease, are these myocardial enzyme indices also related to the severity and prognosis of COVID-19? Therefore, the purpose of this research was to explore the application value of CK, LDH and α-HBDH in myocardial functional injury and acute infectious diseases, and then to evaluate the value of myocardial functional injury in determining the severity and prognosis of COVID-19. This research can provide valuable clinical data for the diagnosis and treatment of COVID-19.

The observation results of this research showed that the average age of the 113 patients was older, most of them were elderly patients over 60 years old. The average age of male patients was higher than that of female patients. Among all the samples, 72 patients were admitted to the hospital with the clinical classification of mild type (male/female: 26/46; <60/≥60: 29/43), and a total of 41 patients (male/female: 27/14; <60/≥60: 11/30) with severe and critically severe type were admitted. According to the difference test, there was a significant statistical difference between the gender of the patients and the clinical classification of admission (p < 0.01). Among the outcomes of the patients, 94 patients (male/female: 40/54; <60/≥60: 38/56) were discharged and improved and 19 patients died (male/female: 13/6; <60/≥60: 2/17). There was a significant statistical difference between different age groups and the outcomes of the patients (p < 0.01). Relevant studies have shown that the elderly are more likely to develop into severe and critical cases [16,17]. This may be related to the fact that the COVID-19 virus attacks the myocardium, patients with underlying diseases and the elderly have a weaker immune system against the pathogen. However, the focus of this study was on the relationship between LDH, HBDH, CK and clinical typing, outcome, disease severity and disease progression.

LDH, α-HBDH and CK had a good judgment on the clinical classification and disease outcome of patients upon admission, and there were significant statistical differences between these three indicators and clinical classification and disease outcome after differential test. In the clinical classification, 28 cases of 113 patients were outside the normal reference range in the first LDH test, including 11 cases of severe patients, 13 cases of critically severe patients and 11 cases of death in the outcome. There were 31 cases of abnormal α-HBDH patients, all above the normal reference range, among which 13 were severe patients, 13 were critically severe patients and 11 patients eventually died. There were 38 abnormal patients with CK, including nine severe patients, 10 critically severe patients and eight patients died. LDH, α-HBDH and CK increased with the deterioration of clinical classification and disease outcome. Our analysis showed that LDH, α-HBDH and CK were significantly increased in different clinical types and outcomes. In early detection, the above three indicators can indicate impaired myocardial function, and LDH, α-HBDH and CK are positively correlated with clinical typing. Therefore, it is suggested that some patients with COVID-19 have clinical manifestations of cardiac involvement [16,18,19]. According to the analysis of the current research data, the impact of COVID-19 on the cardiovascular system mainly includes the direct impact of the virus, accompanied by factors such as fever, hypoxia, inflammation and shock on the cardiovascular system. The bulletin of the American College of Cardiology points out that viral infection can lead to the instability of chronic cardiovascular disease, which is mainly caused by the vigorous metabolism of the body after infection, which increases the burden on the heart and reduces the cardiac reserve, especially the increased risk of acute events or deterioration in patients with coronary heart disease and heart failure [20].

There was a significant positive correlation between LDH, α-HBDH, CK and clinical typing at admission. Spearman correlation analysis showed that the correlation coefficients between LDH, α-HBDH, CK and clinical typing were 0.54, 0.49 and 0.44, respectively. It can be seen that LDH, α-HBDH and CK can be monitored to assess the degree of the patient’s condition. The change of LDH, α-HBDH and CK values can reflect the severity and the trend of the patient’s condition. The linear relationship between LDH, α-HBDH, CK and the outcome was not significant. For severe/critical cases of infection, the inflammatory storm caused by the virus attacking the immune system can cause myocardial and systemic multi-organ damage; In addition, infection can also directly infect the myocardium and lead to injury [21,22]. If myocardial injury is further aggravated due to the combination of underlying diseases, the risk of death is extremely high. Continuous monitoring of LDH, α-HBDH and CK can warn of the deterioration of the disease to a certain extent, whether accompanied by cardiovascular disease or not.

In this research, death and nondeath outcomes were used as the basis for positive classification. The results of ROC curve analysis showed that LDH, α-HBDH and CK had a good diagnostic value for the death outcome of the patient’s end point. The AUC of the three indicators were 0.700 (95% CI: 0.536∼0.864; p < 0.01), 0.650 (95% CI: 0.476∼0.824; p < 0.05) and 0.676 (95% CI: 0.536∼0.817; p < 0.05), are all greater than 0.7. It can be seen that the detection of indicators such as LDH, α-HBDH, CK in patients during clinical treatment has a good diagnostic accuracy for the death outcome. A research found that ACE2 is a receptor for COVID-19 [23], it is a necessary target for coronavirus to infect human body and cause lung damage [24]. However, there is no further direct evidence of whether COVID-19 attacks the cardiovascular, kidney and other organs through ACE2. Whether patients with cardiovascular disease have a higher risk of infected COVID-19 needs further study to determine. In this research, chest CT images of a typical patient at three periods (admission, the 3rd day of hospitalization and the end point) were selected, and the imaging conclusions suggested that the patient had right lung infection and associated atherosclerotic lesions. The dynamic changes of LDH, α-HBDH and CK values associated with myocardial injury were consistent with the results of chest CT.

To sum up, indicators such as LDH, α-HBDH and CK can play an important guiding role in the differentiation of COVID-19 patients’ conditions and the prediction of death outcome. Considering the virus on the body of each system, comprehensive influence, therefore, in the process of diagnosis and treatment should be dynamic monitoring the change of the patients with LDH, α-HBDH, CK and make corresponding prevention and early intervention, especially combined cycle system disease/diagnosis of suspected patients, to some extent, which can reduce the mild/ordinary-to-severe/critically severe patients with deteriorating risk and slow disease progression.

**Summary points**

- The aim was to explore the application value of CK, LDH and α-HBDH in myocardial function injury and acute infectious diseases, and to evaluate the value of myocardial function injury in the severity and prognosis of COVID-19.
- In this research, the values of LDH, α-HBDH, and CK were significantly increased in severe and critically ill patients and patients who died.
- Through receiver operating characteristic curve analysis, LDH, α-HBDH and CK have good diagnostic value for the death outcome of patients.
- LDH, α-HBDH and CK were increased in severe, critically and death patients, which important for diagnosed disease differentiation, outcome progression and death outcome of COVID-19 patients.

## References

[1] Aihua J, Lin J, Benyong Y Clinical features of 19 severe cases of COVID-19 in Beijing[J]. Chin. J. Exp. Clin. Virol. 34(03), 225–230 (2020).

[2] Chaolin H, Yeming W, Xingwang L Clinical features of patients infected with 2019 novel coronavirus in Wuhan, China [published correction appears in *Lancet*. 2020 Jan 30;]. Lancet 395(10223), 497–506 (2020). 31986264 10.1016/S0140-6736(20)30183-5 PMC7159299

[3] Nanshan C, Min Z, Xuan D Epidemiological and clinical characteristics of 99 cases of 2019 novel coronavirus pneumonia in Wuhan, China: a descriptive study. Lancet 395(10223), 507–513 (2020). 32007143 10.1016/S0140-6736(20)30211-7 PMC7135076

[4] Zhongliang W, Bohan Y, Qianwen L, Lu W, Ruiguang Z. Clinical features of 69 cases with coronavirus disease 2019 in Wuhan, China. Clin. Infect. Dis. 71(15), 769–777 (2020). 32176772 10.1093/cid/ciaa272 PMC7184452

[5] Tomasoni D, Italia L, Adamo M COVID-19 and heart failure: from infection to inflammation and angiotensin II stimulation. Searching for evidence from a new disease. Eur. J. Heart Fail. 22(6), 957–966 (2020). 32412156 10.1002/ejhf.1871 PMC7273093

[6] Wang D, Hu B, Hu C Clinical characteristics of 138 hospitalized patients with 2019 novel coronavirus-infected pneumonia in Wuhan, China. JAMA 323(11), 1061–1069 (2020). 32031570 10.1001/jama.2020.1585 PMC7042881

[7] Guan WJ, Ni ZY, Hu Y Clinical characteristics of coronavirus disease 2019 in China. N. Engl. J. Med. 382(18), 1708–1720 (2020). 32109013 10.1056/NEJMoa2002032 PMC7092819

[8] Tadic M, Cuspidi C, Mancia G, Dell'Oro R, Grassi G. COVID-19, hypertension and cardiovascular diseases: should we change the therapy? Pharmacol. Res. 158, 10496–10496 (2020). 10.1016/j.phrs.2020.104906 PMC7217779 32461198

[9] General Office of the National Health Commission, National Administration of Traditional Chinese Medicine. Diagnosis and Treatment of COVID-19 (Trial Version 7)[EB / OL]. (2020). www.gov.cn/zhengce/zhengceku/2020-03/04/content_5486705.htm

[10] General Office of the National Health Commission, Office of National Administration of Traditional Chinese Medicine .“Diagnosis and treatment plan for severe and critical COVID-19 cases (Trial Version Ⅱ)” was released [EB/OL]. (2020). www.gov.cn/zhengce/zhengceku/2020-04/01/content_5497892.htm

[11] Gaze DC. Clinical utility of cardiac troponin measurement in COVID-19 infection. Ann. Clin. Biochem. 57(3), 202–205 (2020). 32255359 10.1177/0004563220921888 PMC7364775

[12] Wu C, Hu X, Song J Heart injury signs are associated with higher and earlier mortality in coronavirus disease 2019 (COVID-19). medRxiv (2020) (Epub ahead of print).

[13] Ndrepepa G, Kastrati A. Creatine kinase myocardial band – a biomarker to assess prognostically relevant periprocedural myocardial infarction. Int. J. Cardiol. 270, 118–119 (2018). 30054147 10.1016/j.ijcard.2018.07.077

[14] Khan AA, Allemailem KS, Alhumaydhi FA, Gowder SJT, Rahmani AH. The biochemical and clinical perspectives of lactate dehydrogenase: an enzyme of active metabolism. Endocr. Metab. Immune Disord. Drug Targets 20(6), 855–868 (2020). 31886754 10.2174/1871530320666191230141110

[15] Yu H, Han H, Li J, Li D, Jiang L. Alpha-hydroxybutyrate dehydrogenase as a biomarker for predicting systemic lupus erythematosus with liver injury. Int. Immunopharmacol. 77, 105922 (2019). 31669891 10.1016/j.intimp.2019.105922

[16] Lian J, Jin X, Hao S Analysis of epidemiological and clinical features in older patients with corona virus disease 2019 (COVID-19) out of Wuhan. Clin. Infect. Dis. 71(15), 740–747 (2020). 32211844 10.1093/cid/ciaa242 PMC7184356

[17] Li X, Xu S, Yu M Risk factors for severity and mortality in adult COVID-19 inpatients in Wuhan. J. Allergy Clin. Immunol. 146(1), 110–118 (2020). 32294485 10.1016/j.jaci.2020.04.006 PMC7152876

[18] Chen L, Li X, Chen M, Feng Y, Xiong C. The ACE2 expression in human heart indicates new potential mechanism of heart injury among patients infected with SARS-CoV-2. Cardiovasc. Res. 116(6), 1097–1100 (2020). 32227090 10.1093/cvr/cvaa078 PMC7184507

[19] Liu PP, Blet A, Smyth D, Li H. The science underlying COVID-19: implications for the cardiovascular system. Circulation 142(1), 68–78 (2020). 32293910 10.1161/CIRCULATIONAHA.120.047549

[20] Jeffrey S. ACC guidance on cardiac implications of coronavirus. www.medscape.com/viewarticle/925244?src=rss

[21] Mehta P, McAuley DF, Brown M COVID-19: consider cytokine storm syndromes and immunosuppression. Lancet 395(10229), 1033–1034 (2020). 32192578 10.1016/S0140-6736(20)30628-0 PMC7270045

[22] Henderson LA, Canna SW, Schulert GS On the alert for cytokine storm: immunopathology in COVID-19. Arthritis Rheumatol. 72(7), 1059–1063 (2020). 32293098 10.1002/art.41285 PMC7262347

[23] Z Z, Yu Zhao, Yujia Wang Single-cell RNA expression profiling of ACE2, the putative receptor of Wuhan 2019-nCov. BioRxiv (2020) (Epub ahead of print).

[24] Peng Z, Xinglou Y, Xianguang W Discovery of a novel coronavirus associated with the recent pneumonia outbreak in humans and its potential bat origin. BioRxiv (2020) (Epub ahead of print).

